# What gastroenterologists should know during COVID-19 Pandemic!

**DOI:** 10.12669/pjms.36.COVID19-S4.2651

**Published:** 2020-05

**Authors:** Lubna Kamani

**Affiliations:** Lubna Kamani, MBBS, FCPS, MRCP (UK), FRCP (London), FACG Associate Professor and Director GI Residency Program, Department of Gastroenterology, Liaquat National Hospital, Karachi, Pakistan

**Keywords:** Corona virus, Gastrointestinal, Liver, COVID-19, Endoscopy

## Abstract

The WHO has declared a Pandemic due to Novel Corona virus-19 (COVID-19). Patients usually have respiratory symptoms but gastrointestinal and hepatic dysfunction are not uncommon presentations and can lead to a delay in diagnosis and management. Virus shedding can continue even after the nasopharyngeal swab gets negative and could lead to faecal-oral transmission. The effects of COVID-19 on patients with decompensated liver disease is still not clear. Managing immunosuppressive drugs in liver transplant and inflammatory bowel disease is a major challenge without clear guidelines. Only emergency endoscopy is to be done with personal protection equipment. Chloroquine and Hydroxychloroquine has shown some beneficial effects and is being used off-label. Without effective treatment, it is imperative to take precautionary measures.

On 11^th^ March 2020, the World Health Organization (WHO) declared a Pandemic due to Novel Corona virus-19 (COVID19).^1^ It is enveloped RNA virus named severe acute respiratory syndrome (SARS-CoV-2), reported from Wuhan, China.^2^ The virus has an incubation period of four to five days (maximum 14 days). Currently more than 200 countries are affected by this virus up till now and Pakistan is yet to see its peak. The government is making all efforts to flatten the curve which will reduce morbidity and mortality. Most commonly patients have a fever and respiratory symptoms which lead to multi-organ failure in some cases.^3^ Digestive symptoms like nausea, vomiting, abdominal pain and diarrhea are not uncommon in COVID-19 patients and can be seen in up to 10% of the cases.^4^ There has been case reports of patient presenting with hemorrhagic colitis^5^ and acute hepatitis^6^ as the first symptom. These a typical presentation can lead to delay in diagnosis and management.

The virus gains entry via Angiotensin Converting Enzyme 2 (ACE2) receptors abundantly present in cilia of glandular epithelium in gastrointestinal tract and cholangiocytes.^7^ Definite mechanism of hepatic dysfunction is yet to be defined but it has been proposed that it could be a direct virus effect on hepatocytes or secondary to inflammation, immune mediated or drug toxicity.^8^ As active viral replication occurs in small and large intestine, there is ample evidence that SARS-CoV-2 RNA is found in stool samples and anal swabs. It has been suggested that virus shedding can continue up to 30 days in stool even after resolution of symptoms and negative nasopharyngeal swab.^9^ This observation can lead to major change in clinical policies of incorporating anal/rectal swab or stool testing before discharging these patients as it might be a source of faecal-oral transmission in community.

The effect of COVID-19 on immunosuppressed patients like advanced chronic liver disease, liver transplant and patients with inflammatory bowel disease on biologics is still not well established. Patients on immunosuppressive medications without COVID-19 need close surveillance without routine reduction of these medications, whereas those with severe COVID 19 infection may need to consider adjusting their dosage of steroids, calcineurin inhibitor or mycophenolate. All potential donors and recipient should be screened for COVID-19.^10^

As there is no cure, prevention is the key. Frequent hand washing with soap and water for at least 20 seconds, avoid touching your face and eyes, social distancing and good nutrition are amongst a few instructions given by the WHO. Till date there is no effective vaccine for COVID-19 and trials of passive immunization are underway.^11^ There is some initial data regarding efficacy of Choroquine (CQ), Hydroxycholoroquine (HCQ) and azithromycin in these patients and clinicians across the globe are involved in its off label usage. However, because of potential fatal side effects of CQ and HCQ one has to be cautious.^12^ Recently FDA has issued emergency authorization of CQ and HCQ to treat COVID patients. The WHO is also conducting a large multicenter five arm solidarity trial comparing four regimes: remdesevir, CQ, lopinavir– ritonavir, and lopinavir–ritonavir plus interferon beta, all compared with standard of care treatment.^13^ There is some initial evidence that BCG vaccine might provide some protection against COVID-19.^14^

According to the American Gastroenterology Association (AGA)^15^, endoscopic procedures are considered high risk because it is an aerosol generating procedure. Elective procedures need to be rescheduled and only emergency procedures like Upper gastrointestinal bleed (GI), cholangitis, foreign body impaction and stenting to relieve GI obstruction should be performed. Doctors and staff involved in procedure should wear N95 mask, double gloves and proper personal protection equipment (PPE), whereas negative pressure rooms (where available) are to be preferred over routine endoscopy rooms in confirmed or suspected COVID-19 patients. All endoscopes should undergo regular disinfection process.

## CONCLUSION

Gastrointestinal and liver dysfunction is not uncommon in patients with COVID-19 infection and it may occur without respiratory symptoms, high index of suspicion is required for early diagnosis and management. In the absence of effective treatment, it is imperative to take all precautionary measures for its prevention.
